# Editorial: Mineral nutrients on tea yield and quality formation

**DOI:** 10.3389/fpls.2023.1192432

**Published:** 2023-05-15

**Authors:** Lili Sun, Zhaoliang Zhang, Yeyun Li, Jianyun Ruan, Tanmoy Karak, Tianyuan Yang

**Affiliations:** ^1^ State Key Laboratory of Tea Plant Biology and Utilization, School of Tea and Food Science and Technology, Anhui Agriculture University, Hefei, China; ^2^ Root Biology Center, Fujian Agriculture and Forestry University, Fuzhou, China; ^3^ Tea Research Institute, Chinese Academy of Agriculture Sciences, Key Laboratory of Tea Biology and Resource Utilization of Tea, The Ministry of Agriculture, Hangzhou, China; ^4^ Upper Assam Advisory Centre, Tea Research Association, Dibrugarh, Assam, India

**Keywords:** *Camellia sinensis* (L.) O. Ktze, mineral nutrients, tea yield and quality formation, fertilizer, efficient use of nutrients

Mineral nutrients are the food of plants. The absorption and utilization efficiency of nutrients are crucial for improving the yield and quality of tea. Tea plant is an economically important leaf crop. Long-term harvesting and pruning can lead to a significant deficiency of nutrients, causing a decline in tea plant vigor and a severe decrease in tea yield and quality. Furthermore, tea plants adapt to acid soil, in which the nutrients are easily to be lost. In order to pursue higher tea yields, a large amount of fertilizer is usually used in tea plantation, leading to a series of environmental problems such as soil compaction. However, current research is mainly focused on the quality of tea processing in the field of tea, with little attention paid to mineral nutrients. This special issue mainly studies the influence of mineral nutrients on tea yield and quality, and includes four research articles covering soil nutrient characteristics, the impact of mineral nutrients on tea yield and metabolites, and the effect of pruning on the distribution of mineral nutrients.

The growth of tea plant adapts to acidic soil. Through physical and chemical analysis of 7300 soil samples from 115 tea gardens in India, Malakar et al. found that more than half of the pH values of tea plantation soils in the region were between 4.5-5.5, which is suitable for the growth of tea plants. Nitrogen is an important macronutrient for plants, especially for tea, which requires a large amount of nitrogen due to frequent harvesting and pruning. The autophagy process of plants can improve the utilization efficiency of mineral nutrients. Huang et al. screened *CsATG101* gene in tea trees, and found that the expression level of this gene in most mature leaves was higher than that in young leaves among 24 tea tree varieties. This suggests that the autophagy process is stronger in mature leaves. This gene might be used as a candidate gene to increase the yield of fresh leaves under different nitrogen levels. In addition, tea plants prefer ammonium nitrogen, but its regulation mechanism for tea tree growth is unknown. Wang et al. treated tea trees with ammonium nitrogen deficiency and recovery, and found that ammonium nitrogen may act as a signaling molecule affecting catechins and thus affecting growth. However, there is also a concentration range of ammonium nitrogen, otherwise, tea plants will be stressed under high ammonium conditions. Pruning is an important strategy to improve the yield of fresh leaves in tea garden management. Liu et al. compared tea leaves from 1962 pruned and 1188 unpruned tea plants, and found that pruning significantly reduced the concentration of easily mobile elements such as nitrogen, phosphorus, potassium, and magnesium, while significantly increased the concentration of immovable elements such as calcium and aluminum. Additionally, calcium and magnesium contributed the most to the differences in metabolites, providing a management improvement measure to improve the quality of tea.

In conclusion, the research in this topic highlights the importance of mineral nutrients and their impact on tea production and quality. The findings suggest that improving mineral nutrient management in tea cultivation can lead to better tea quality and production.

## Author contributions

All authors listed have made a substantial, direct, and intellectual contribution to the work and approved it for publication.

